# The intrinsic reason why complementary tests (clinical neurophysiology, neuroimaging, skin biopsy) cannot establish the diagnosis of neuropathic pain

**DOI:** 10.3389/fpain.2025.1723124

**Published:** 2026-01-15

**Authors:** Jean-Pascal Lefaucheur

**Affiliations:** 1 UR4391 (ENT Team), Faculty of Health, Paris Est Créteil University, Créteil, France; 2 Department of Clinical Neurophysiology, Henri Mondor University Hospital, AP-HP, Créteil, France

**Keywords:** clinical neurophysiology, diagnosis, evoked potentials, magnetic resonance imaging, neuropathic pain, skin biopsy

## Abstract

Neuropathic pain is defined as pain caused by a lesion or disease of the somatosensory nervous system. Current algorithms for neuropathic pain diagnosis include patient history, clinical examination, and complementary tests to confirm a lesion or disease of the somatosensory nervous system, able to change the diagnosis of neuropathic pain from probable to definite. These tests include clinical neurophysiology, such as pain-related evoked potentials, quantitative sensory testing, skin biopsy to measure intraepidermal nerve fiber density, or magnetic resonance imaging. However, these tests are especially relevant to demonstrate a structural lesion of the somatosensory system leading to sensory deficit, but they cannot establish a causal link between nervous lesion and the presence of pain. Similar lesions of the somatosensory nervous system may be accompanied by pain or not, while neuropathic pain can be a matter of sensitization or hyperexcitability of somatosensory structures without overt structural lesion. Even the existence of hyperexcitability of nociceptive pathways, revealed by neurophysiological or genetic tests, may contribute to the emergence of pain, but may not be sufficient to affirm that this results in ongoing neuropathic pain. Thus, various complementary tests can be useful to identify a lesion of the somatosensory nervous system, but not to confirm the presence of associated neuropathic pain. Clinical assessment, considering disease history, symptom descriptors and a plausible neuroanatomical distribution, remains the cornerstone of the diagnosis of neuropathic pain, while paraclinical findings must be interpreted with caution in this regard.

## Introduction: definition and diagnosis of neuropathic pain

In 1994, the Task Force on Taxonomy of the International Association for the Study of Pain (IASP) introduced the concept of “neuropathic pain”, defined as “pain initiated or caused by a primary lesion or dysfunction in the nervous system” (1). In 2008, a group of experts proposed a new definition of neuropathic pain, as “pain arising as a direct consequence of a lesion or disease affecting the somatosensory system” (2), by replacing the terms “dysfunction” and “nervous system” with “disease” and “somatosensory system”, respectively. In this article, a diagnostic workup algorithm was also proposed, comprising a first step based on patient interview, in particular to collect a medical history “suggestive of a relevant lesion or disease affecting the peripheral or central somatosensory system” and to confirm that pain has “a distinct neuroanatomically plausible distribution”; a second step based on clinical examination, in particular to look for “negative or positive sensory signs, confined to innervation territory of the lesioned nervous structure”; a third step based on complementary diagnostic tests, including neurophysiology, neuroimaging, and biopsy, to “confirm lesion or disease explaining neuropathic pain” (2). The positivity of these tests was mentioned as required to establish a definite diagnosis of neuropathic pain in this “grading system” (2).

In 2011, the IASP Terminology Working Group endorsed the definition of neuropathic pain as “pain caused by a lesion or disease of the somatosensory nervous system” (3). In 2018, the “grading system” for the diagnosis of neuropathic pain was updated (4). This article has broadly retained the same prior algorithm, but introducing the use of neuropathic pain questionnaires as screening tools, specifying some clinical elements on the “neuroanatomically plausible distribution” of pain symptoms and further emphasizing that “reaching the final level of certainty (definite neuropathic pain) requires that an objective diagnostic test confirms the lesion or disease of the somatosensory nervous system”. Finally, following two previous publications produced in 2004 and 2010 by a working group of the European Federation of Neurological Societies (5, 6), the guidelines on the assessment of neuropathic pain were updated in 2023 by a joint group of the European Academy of Neurology (EAN), the European Pain Federation (EFIC) and the IASP Neuropathic Pain Special Interest Group (NeuPSIG) (7). In these EAN-EFIC-NeuPSIG guidelines, the complementary tests used for the diagnosis of neuropathic pain were reviewed. A strong recommendation was proposed for the use of skin biopsy and a weak recommendation for quantitative sensory testing and nociceptive evoked potentials. Additionally, a strong recommendation was also proposed for trigeminal reflex recording and genetic testing supporting their usefulness in selected cases (7).

The major and intrinsic problem of all these algorithms and recommendations previously published is the question of the causality of the anomalies highlighted by the complementary tests to explain neuropathic pain. The value, specificity, and even sensitivity of these tests in demonstrating structural lesions of peripheral or central nervous structures belonging to the somatosensory system are undeniable. By definition, “lesion” is “a generic term used in medicine to designate any alteration in the anatomical or histological characteristics of an organ”. In the context of the somatosensory nervous system, “structural lesions” consist of morphological abnormalities, which may be visible histopathologically, such as nerve fiber loss, axonal degeneration/regeneration, or demyelination. Such structural lesions, possibly identified by complementary tests, can only be considered as intrinsically causal of negative symptoms or signs, i.e., of clinical sensory deficit.

Conversely, it is not inherently possible to attribute a direct causal link between a structural lesion identified by these tests and the existence of pain. Indeed, regardless of the neurological lesion revealed by these tests, it may or may not be accompanied by pain. Consequently, the involvement of a neurological lesion in a painful manifestation can only be speculative, based on clinical considerations (disease history, specific descriptors of neuropathic pain, and a plausible neuroanatomical distribution) and not on the actual identification of the lesion.

On the other hand, neuropathic pain is not always linked to a structural lesion of the somatosensory nervous system, but may be related to a disease that only produces neural activity (functional) changes affecting the nerve fibers or pathways of the somatosensory system and the transmission of information at the level of these fibers or pathways. These “functional changes” involve alterations in the activity (essentially a gain of function or hyperexcitability) of axonal ion channels or synaptic transmission, leading to neuropathic pain without histopathological hallmark.

Indeed, neuropathic pain related to functional changes in neuronal excitability in the absence of structural neuropathological lesions involves various types of voltage-gated ion channels (8), which play a crucial role in modulating resting neuronal excitability as well as signal transmission along axons and sensory afferent pathways. These changes contribute to aberrant neuronal activation, the generation of ectopic discharges, the dysregulation of neurotransmitter release, and pain sensitization. Thus, various situations of neuronal hyperexcitability promote the development and maintenance of a persistent neuropathic pain state (8).

## Objective and methodology

This article offers a conceptual analysis aimed at questioning the role of complementary tests, which have been previously discussed and proposed for the diagnosis of neuropathic pain (7). In this theoretical article, the evidence provided by these tests is reviewed, demonstrating that while they are relevant for demonstrating “structural lesions” of the somatosensory nervous system, they cannot establish a direct causal link with the existence of neuropathic pain associated with a lesion or disease of this system. The issue of central sensitization is also addressed, as it can be secondary to neuropathic pain. However, this phenomenon is not specific: central sensitization contributes, in particular, to the chronicity of pain, either initially nociceptive or primary, thus defining nociplastic pain. In this text, the diagnostic tests analyzed were selected based on previous recommendations (7). The arguments are supported by examples drawn from existing literature (PubMed/Medline) or personal observations.

## Complementary tests used to assess neuropathic pain

### Clinical neurophysiology

**Figure 1:**
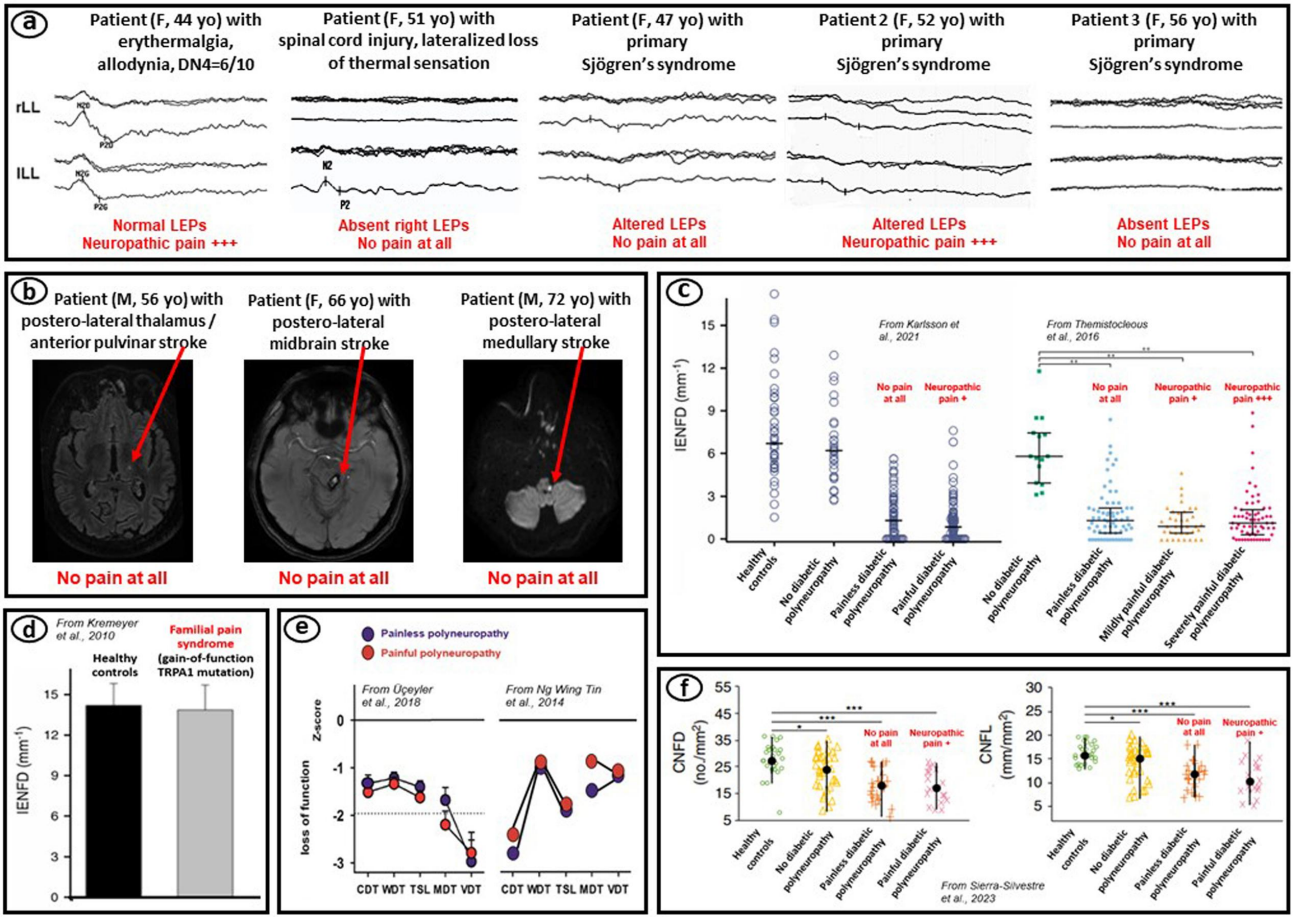
Examples illustrating the lack of relevance of complementary tests to demonstrate the presence of neuropathic pain. **(a)** laser evoked potentials (LEPs) (personal unpublished data); **(b)** magnetic resonance imaging of the brain (personal unpublished data); **(c,d)** skin biopsy with measurement of intraepidermal nerve fiber density (IENFD) [adapted from Karlsson et al. (10), Themistocleous et al. (11), and Kremeyer et al. (12)]; **(e)** quantitative sensory testing with measurement of cold detection threshold (CDT), warm detection threshold (WDT), thermal sensory limen (TSL), mechanical detection threshold (MDT), vibration detection threshold (VDT) [adapted from Üçeyler et al. (13) and Ng Wing Tin et al. (14)]; **(f)** corneal confocal microscopy with measurement of corneal nerve fiber density (CNFD) and corneal nerve fiber length (CNFL) [adapted from Sierra-Silvestre et al. (15)]. F, M: female, male patient; yo, years old.

Regarding clinical neurophysiology, the recommended tests are those investigating the pain pathways (7), such as pain-related evoked potentials (PREPs) to nociceptive stimuli, using either thermal stimuli (laser evoked potentials or contact-heat evoked potentials) or electrical stimuli (intraepidermal electrically evoked potentials) (9). However, PREPs can be altered, delayed, or absent in case of fiber loss or loss of function of either small-diameter nerve fibers or spinothalamic tract fibers, regardless of the presence of concomitant pain as illustrated in Figure 1.

In fact, a loss of function affecting pain pathways is directly responsible for a thermoalgesic sensory deficit, and not for neuropathic pain, which is instead linked to the hyperactivity or hyperexcitability of these pathways. Such hyperexcitability could be revealed by PREPs of increased amplitude. However, increased PREPs are rather observed in cases of central sensitization of brain activities associated with nociplastic pain, such as in fibromyalgia, rather than neuropathic pain (16–18). Nociplastic central sensitization affecting either sensory or attentional aspects of pain can lead to increase PREP amplitude (19). Conversely, patients with hyperalgesia, but associated with peripheral or central neurological injury, may have impaired or absent PREPs due to the lesion (20). In summary, altered PREPs are relevant and very useful for demonstrating a lesion of the pain and temperature pathways at the peripheral, spinal, or supraspinal level, but not the presence of pain itself. Enhanced PREPs may eventually reveal pain-related sensitization (hyperalgesia), but without specificity as to the type of sensitization, either neuropathic, at the peripheral or central somatosensory level, or nociplastic, at the level of cerebral pain matrices.

Trigeminal reflexes have also been highlighted as being useful for the diagnosis of trigeminal neuropathic pain (7). In fact, neurophysiological recording of trigeminal reflexes, as well as trigeminal PREPs, is certainly very sensitive in demonstrating injury to trigeminal nerve afferents, or its nuclei or pathways in the brainstem (21, 22). However, again, such injury may be associated with pain or not, and the injury itself is not predictive of the presence of pain, i.e., painful trigeminal neuropathy. Regarding trigeminal neuralgia, the key pathophysiological mechanism is trigeminal nerve hyperexcitability, either primary, at the brainstem entry zone, related to microvascular conflict in classic trigeminal neuralgia, or secondary, at the nucleus level, related to brainstem injury in patients with multiple sclerosis for example (23). Unfortunately, nerve hyperexcitability can only be detected by neurophysiological tests used in experimental or research studies, and not in routine clinical practice (9). Therefore, standard clinical neurophysiological test cannot identify neuralgia, which is related to hyperexcitability rather than nerve fiber loss.

Another illustrative example is pudendal neuralgia linked to pudendal nerve compression. The diagnosis of pudendal neuralgia is only based on clinical criteria (24), while electrodiagnostic tests of the pudendal nerve are useless or even misleading (25). Particularly in the female population depending on age and surgical, digestive or gynecological history, the rate of pudendal nerve injury is high, which leads to a high probability of finding corresponding neurophysiological abnormalities. On the other hand, the frequency of pelvic nociplastic pain is high in this population (26). Also, for statistical reasons, it is common to observe neurophysiological alterations of the pudendal nerve in an elderly woman that are neither causal nor involved in a pelvic pain syndrome, which is in fact of nociplastic origin. On the other hand, simple compressive mechanical irritation of the pudendal nerve can be the cause of pain, but without causing neurophysiological alterations. Therefore, electrodiagnosis has been excluded from the diagnostic work-up of pudendal neuralgia related to pudendal nerve compression syndrome, as being neither sensitive nor specific (27).

However, some neurophysiological tests are able to show hyperexcitability or sensitization of noxious afferents or nociceptive information transmission in humans, such as microneurography (28, 29) at peripheral level or RIII reflex [nociceptive flexion (withdrawal) reflex] recording (30, 31) at spinal level. These tests have the indisputable advantage of being directly linked to certain pain mechanisms rather than to the detection of neurological lesions. However, their implementation is impractical in routine clinical settings. Moreover, these tests rely primarily on the quantification of responses to noxious stimuli. Therefore, these recordings are likely more relevant for assessing symptoms of evoked pain than those of ongoing spontaneous pain. In other words, the hyperexcitability of nociceptive fibers highlighted by these provocation tests cannot predict the presence of spontaneous neuropathic pain.

Central sensitization causing evoked pain can be assessed by neurophysiological tests. For example, there are standardized models of secondary hyperalgesia in humans with spinal sensitization assessed by recording the RIII reflex, the spinal N13 component of upper limb somatosensory evoked potentials, or pinprick-evoked potentials (32, 33). These approaches are rather experimental and not clinically relevant for the diagnosis of neuropathic pain. The same applies to the use of neurophysiological techniques (mainly RIII) in temporal summation of pain (TSP) procedures that aim to assess the proalgesic spinal wind-up phenomenon involving wide dynamic range neurons of the dorsal horn (34, 35). Various electrophysiological techniques are also used in conditioned pain modulation (CPM) procedures (36) that assess analgesic diffuse noxious inhibitory control (pain-inhibits-pain phenomenon), but whose alteration is not specifically involved in the development of neuropathic pain and does not allow its diagnosis.

In fact, only microneurographic recordings of spontaneous activity of sensory nerve fibers can truly provide information directly related to ongoing neuropathic pain, at least at peripheral level. This goal has been pursued by several studies performed in humans with painful neuropathy (37–42). However, due to the very demanding electrophysiological technique required to measure spontaneous activity of sensory afferents, these results exhibit high variability and uncertainty (43). In addition, this technical approach has limitations in terms of quantification of the observed abnormalities and their clinical relevance in the context of chronic pain syndrome, which does not predict how these markers of peripheral hyperexcitability are integrated at the central level in a multifaceted experience with complex biopsychosocial aspects (44).

Thus, it can be assumed that the relevance of electrophysiological techniques for the diagnosis of neuropathic pain must be based on recordings of spontaneous activities and not based on responsiveness to sensory stimuli. At the peripheral nerve level, microneurography could meet this objective, with the limitations we have mentioned. At the spinal level, unfortunately, to our knowledge, there is no possibility of non-invasive recording of dorsal horn activities applicable in clinical practice in living humans. However, invasive recordings are now permitted in the spinal epidural space (45) as well as the intracranial space (46) to potentially characterize chronic pain status with surgically implanted electrodes. At the cerebral level, obviously, spontaneous electrical activities can be recorded by means of resting-state electroencephalography (EEG) or magnetoencephalography (MEG). Resting-state EEG has been relatively little studied in chronic neuropathic pain. A recent review of the literature suggests that EEG signal power is increased in the θ band (4–7 Hz) and possibly in the high-β band (20–30 Hz), but decreased in the high-α-low-β band (10–20 Hz) in the presence of ongoing neuropathic pain (47). However, these features may not be specific to the neuropathic character of chronic pain (48), if indeed there is a neuropathic specificity in brain activity changes associated with pain. In any case, this is an important avenue to find objective biomarkers of chronic neuropathic pain in the future using advanced techniques of EEG signal analysis, including source location (49), functional connectivity in the dynamic pain connectome (50–54), microstate analyses (55), aperiodic component activity (56), or classification methods based on machine-learning and artificial intelligence (57–62). Further perspectives are opened by combining EEG with transcranial magnetic stimulation techniques (63, 64). However, at present, the diagnosis of neuropathic pain cannot be made using any EEG approach.

A final word should be added regarding autonomic nervous system testing. Given the association between dysautonomia and neuropathic pain in different clinical situations, such as painful neuropathies (65, 66), autonomic nervous system tests could be proposed for the assessment of neuropathic pain. However, their causal justification is even more limited than sensory electrophysiological tests in this context; therefore, we will not discuss these tests in this article.

### Quantitative sensory testing

Most quantitative sensory testing (QST) studies have shown no striking differences in sensory detection thresholds between patients with painful or painless polyneuropathy (13, 14, 67–72) or whether focal nerve injury was accompanied by pain or not (73, 74). And although some studies have found increased sensory thresholds in patients with painful neuropathy compared to those with painless neuropathy (11, 75), this finding cannot be simply explained by a “deafferentation pain” mechanism, as there is no evidence of a causal link between an increased sensory detection threshold and the presence of pain. In fact, an increased sensory threshold can only be directly responsible for hypoesthesia at clinical level. From a pathophysiological perspective, an increased sensory threshold may actually indicate greater severity of sensory nerve damage in painful neuropathies, with possible common factors, such as inflammation (76–80), underlying both the severity of nerve injury and neuropathic pain rather than a direct causality between injury and pain.

In fact, it is even the fact that sensitivity is preserved that may explain certain aspects of chronic neuropathic pain. For example, in amyloid neuropathy, lower thermal thresholds, thus indicating a better preservation of small-diameter fibers, were associated with greater intensity of ongoing burning sensations (81). In summary, altered sensory thresholds can indicate the severity of neuropathy (82, 83) but cannot predict neuropathic pain.

QST techniques can also be used to characterize the existence of evoked pain (allodynia or hyperalgesia) by measuring pain detection thresholds or the intensity of pain produced by sensory stimulation at different magnitudes. Although some studies have shown a high rate of allodynia in patients also presenting spontaneous pain related to peripheral neuropathy (11, 14, 84), unfortunately, as previously mentioned, the presence of allodynia or hyperalgesia does not predict the presence of spontaneous neuropathic pain related to hyperexcitability of nociceptive receptors or nerve fibers (72, 75). QST methods are widely used in TSP protocols to assess the wind-up phenomenon in the spinal cord (85), but the diagnostic sensitivity of these protocols is questionable due to significant inter-individual variability (86). A similar conclusion can be made for the use of QST techniques in CPM protocols to assess diffuse noxious inhibitory control (86, 87). In fact, these tests can be used to determine a “pain modulation profile” differentiating an antinociceptive profile (low TSP/high CPM) from a pronociceptive profile (high TSP/low CPM). This approach was proposed to be used in clinical practice to predict the risk of chronic postoperative pain or osteoarthritis-related pain for example (88–90). However, the relevance of QST is also debated in these contexts (91, 92), including in its ability to predict pain level or associated disability on an individual level (93). Thus, in any way, the use of QST, even using complex procedure, can be supported to make the differential diagnosis between neuropathic pain and other types of pain, including secondary central sensitization (94).

QST data have also been used to determine different “sensory profiles” in patients suffering from neuropathic pain. A “binary” classification was initially proposed, distinguishing patients with more or less global hypoesthesia reflecting a loss of function of small and/or large nerve fibers on the one hand, and patients with thermal and/or mechanical hyperalgesia (pain related to “irritable” nociceptors) on the other hand (95). Subsequently, the “comprehensive” QST battery of the German DFNS working group was developed to assess all types of sensory alterations (96, 97). A study based on the analysis of QST examination data performed with this protocol in more than 1,000 patients with neuropathic pain was initiated in Germany (98) and then repeated at the level of a European consortium (99, 100) to lead to the characterization of three distinct “sensory profiles”, particularly in patients with peripheral neuropathies (98): (i) global hypoesthesia (sensory loss), (ii) thermal hyperalgesia, and (iii) mechanical hyperalgesia more or less combined with thermal hypoesthesia. More recently, simplified QST approaches have been proposed for this same objective (101, 102). The three “sensory profiles” thus defined have been interpreted as revealing, respectively, the existence of deafferentation pain, peripheral sensitization, and central sensitization. In a recent study (103), patients suffering from central neuropathic pain secondary to spinal cord injury presented with a profile of sensory loss or mechanical hyperalgesia, with or without associated thermal hyperesthesia, but not hypoesthesia. However, as previously noted, the association between sensory loss and pain phenotype does not necessarily indicate a mechanistic causality between the two phenomena, as hyperesthesia is not directly related to the injury, but to secondary hyperexcitability mechanisms of potentially diverse origins.

In any case, these “sensory profiles” do not have diagnostic value in themselves to define the neuropathic or non-neuropathic nature of a pain syndrome, but could help identify responders to certain specific treatments for neuropathic pain (104). However, the relevance of this QST-guided approach remains open to debate (105).

### Skin biopsy

In clinical practice, the skin biopsy technique is based on the measurement of the intraepidermal nerve fiber density (IENFD) (106). A decrease in IENFD reflects a loss of small-diameter nerve fiber endings and is therefore causally responsible for thermoalgesic cutaneous hypoesthesia, but not necessarily for the concomitant presence of pain. Various studies have shown the high sensitivity of skin biopsy to objectify small fiber neuropathy, but have not shown differences in IENFD values between patients with painful or painless neuropathy (10, 11, 107–112) (Figure 1). Statistical correlations have been observed between fiber loss (reduced IENFD) and pain intensity in some studies (70, 113–115), but this does not demonstrate a causal relationship. Furthermore, correlations remain very weak (107, 116) or absent in most studies (117–121). IENFD values also do not correlate with the intensity of neuropathic pain symptoms, as specifically assessed by the Neuropathic Pain Symptom Inventory (122). Small fiber loss (reduced IENFD) has even been found to be inversely correlated with pain intensity in patients with focal neuropathic pain due to surgical nerve injury (123). Similarly, preservation of IENFD has been associated with the existence of hyperalgesia in patients with peripheral neuropathy (124).

Other skin biopsy variables than IENFD might clearly be more relevant for being associated with the presence of pain in the context of small fiber neuropathy. For example, increased fiber length, branching, or axonal swellings have been suggested to correlate with pain intensity (107, 108), but this finding has been disproved (10, 125). Some protein immunostainings, such as intraepidermal expression of Growth Associated Protein 43 (GAP-43) (109) or Transient Receptor Potential Vanilloid 1 (TRPV1) (126), or dermal expression of Calcitonin Gene-Related Peptide (CGRP) (126) have been associated with some components of neuropathic pain, but not in all studies (127). Finally, pain may be associated with changes in the number of Langerhans or Schwann cells, but conflicting results have also been published regarding these variables (118, 128, 129).

In the context of peripheral neuropathies, the relationship between nerve fiber structure and function is complex (130) and, therefore, the simple counting of IENFD on a skin biopsy cannot constitute relevant evidence of the presence of neuropathic pain. This parameter is primarily an indicator of neurodegeneration, which cannot be intrinsically accepted as causal for the occurrence of neuropathic pain, just like other biomarkers of neurodegeneration, such as the level of neurofilament light chains (131). Another example showing that correlation does not mean causation is the fact that, in peripheral neuropathies, there is a strong correlation between IENFD measured at the ankle et the amplitude of distal sensory nerve action potentials of the sural nerve (116, 132–134), while these two parameters do not evaluate the same nerve fibers at all and of course do not have a direct causal link, but may simply reflect a common severity for different nerve fiber types during the progression of a peripheral neuropathy. In conclusion, the presence of neuropathic pain cannot be proven by a skin biopsy, and certainly not by the measurement of IENFD, contrary to what is suggested by some guidelines (7).

### Corneal confocal microscopy

Corneal confocal microscopy (CCM) allows the measurement of intracorneal nerve fiber density (CNFD). This measure, similar to the IENFD for skin biopsy, is relevant to demonstrate focal loss of small-diameter nerve fibers, potentially indicating the existence of more diffuse small fiber neuropathy (135). However, other measurements have been added to CNFD, such as corneal nerve fiber length (CNFL) or “branching” density (CNBD), to increase the diagnostic sensitivity of CCM examination (136). Some studies have shown a correlation between certain CCM variables, either CNFD, CNFL or CNBD, and the severity of neuropathic pain (108, 137–141), although other studies have not found it (15, 142–145). Based on such a correlation, the use of CNFD reduction has been proposed to identify peripheral neuropathic pain (140). However, correlation does not imply causation, and CNFD reduction only identifies neuropathy (loss of corneal nerve fibers), not the presence of neuropathic pain. In accordance with pathophysiological considerations, these measures cannot establish a causal link between structural lesions of the corneal nerves and the development of pain, including ocular pain.

### Magnetic resonance imaging

Structural magnetic resonance imaging (MRI) could be relevant for the diagnosis of neuropathic pain if certain brain lesions were specific for the presence of pain. For example, two studies showed that patients with central post-stroke pain (CPSP) tended to have posterior thalamic lesions, affecting the posteroinferior regions of the ventroposterolateral nucleus or even more specifically the anterior pulvinar (146, 147). A posterolateral midbrain lesion is another potential cause of CPSP (148). However, the presence of CPSP cannot be definitively confirmed solely by MRI location of a brain lesion, given the heterogeneity of the pathophysiological mechanisms potentially involved in this clinical setting (149, 150). Thus, patients with a stroke lesion in the posterolateral brainstem or thalamic region may not experience pain, as illustrated in Figure 1. Therefore, it is possible that injury to the spinothalamic afferents of the ventrocaudal thalamus increases the risk of developing CPSP, but the occurrence of such pain at the individual level for a given patient is impossible to predict based on the lesion demonstrated by MRI. Structural neuroimaging of lesions of the spine or peripheral nervous system shares the same limitations.

Apart from lesions, structural neuroimaging can also show changes in gray matter volume, cortical thickness, gyrus morphometry, or sulcus depth in comparison between painful and painless neuropathic conditions or in correlation with pain intensity (151–154).

Functional brain imaging, such as functional MRI (fMRI), can provide important pathophysiological information on changes in brain activation in response to painful stimuli, relevant for assessing evoked pain conditions (allodynia and hyperalgesia) or pain relief by analgesic procedures (155). However, at the individual level, functional brain imaging does not currently allow, in clinical practice, to assist in the differential diagnosis between neuropathic pain and another pain condition which may, for example, be associated with central sensitization of brain circuits (nociplastic pain). Furthermore, fMRI is not relevant for understanding spontaneous pain, except by performing “resting state” (rs) imaging.

In fact, rs-fMRI can be used to assess changes in functional connectivity to potentially correlate them with pain severity. Only a few papers assess differences between patients with and without ongoing neuropathic pain in the context of neurological disease or injury, e.g., in the context of painful polyneuropathies (156, 157). Connectivity changes can also be assessed on structural basis using diffusion tractography (158, 159).

Finally, functional differences between patients with painful or painless neuropathic disorders can be demonstrated by magnetic resonance spectroscopy, based on changes in the levels of various biological markers, such as N-acetyl aspartate, glutamate, γ-aminobutyric acid, or phosphocreatine, for example in the thalamus or somatosensory cortex (160–163).

However, regardless of the technique, neuroimaging cannot be used at the individual level to diagnose neuropathic pain, and the search for relevant biomarkers remains a major preliminary objective in this field.

### Genetic studies

Neuropathic pain has been associated with gain-of-function mutations in skin nociceptors (TRPA1) (12) or axonal membrane sodium channels (NaV1.7–1.9 corresponding to genes SCN9A–11A) (164–166), involved in the propagation of action potentials in small-diameter nociceptive nerve fibers. A gain-of-function mutation can lead to hyperexcitability of these nociceptive fibers causing pain, but cannot causally explain a “lesional” small fiber neuropathy unless the intra-axonal influx associated with hyperexcitability leads to neuronal excitotoxicity, which is a pathological process that remains to be demonstrated (167). Thus, the concept of a gain-of-function mutation as a cause of idiopathic small fiber neuropathy, as evidenced by fiber loss on skin biopsy, is difficult to support, despite several articles highlighting the presence of this association in up to 30% of patients with idiopathic small fiber neuropathy (168–170).

On the other hand, a number of gain-of-function sodium channel variants (such as the R1150W isoform, present in approximately 10% of the general population), do not necessarily lead to the presence of spontaneous neuropathic pain (171). Most studies are limited to showing the presence of a gain-of-function variant in pain patients, without looking for the absence of this variant in non-painful relatives, the only element that can demonstrate a specific implication of a given sodium channel mutation in pain. Thus, even the presence of a gain-of-function of nociceptive receptors or axons is not necessarily causal for the occurrence of spontaneous neuropathic pain. Some gain-of-function mutations probably only represent a cofactor promoting the emergence of neuropathic pain under the influence of other acquired, metabolic, toxic or immuno-inflammatory factors. In reality, gain-of-function mutations that are truly pathogenic and directly responsible for familial neuropathic pain syndromes, mainly characterized as inherited erythromelalgia or paroxysmal extreme pain disorder (172, 173), probably remain very rare.

## Discussion

Alterations in PREPs, QST, or structural MRI, as well as reductions in IENFD or CNFD, are certainly sensitive and relevant factors for demonstrating the existence of damage to thermal or nociceptive somatosensory pathways. However, structural lesions of small-diameter sensory nerve fibers or spinothalamic tracts result in thermoalgesic sensory loss and are not predictive of the presence or absence of associated spontaneous neuropathic pain.

Numerous examples illustrate this conclusion. For example, patients with postherpetic neuralgia (PHN) may experience burning pain sensations related to abnormal peripheral sensitization of unmyelinated cutaneous nociceptors (irritable nociceptors) without significant sensory loss (95). Other patients with PHN present with severe sensory deficits, where dorsal root ganglion neuronal loss is presumed. In this case, persistent burning pain could arise from abnormal spontaneous hyperactivity of second-order dorsal horn neurons, deprived of primary afferent connections, and/or reorganization of central connections (95). However, sensory loss is not the intrinsic cause of pain and does not always result in pain. Other patients with PHN exhibit mechanical allodynia, likely due to the formation of new connections between functionally or structurally altered large-diameter non-nociceptive primary afferents and central pain-transmitting neurons (95). Neither persistent hyperactivity of peripheral axons or dorsal horn neurons nor the resulting changes in synaptic plasticity can be demonstrated by complementary tests, not even microneurography or neurophysiological tests based on stimulation techniques. These tests can only support the presence of allodynia or hyperalgesia, and not primarily the diagnosis of neuropathic pain.

Strong evidence suggests that spontaneous activity, such as ectopic activity and high-frequency discharges, occurring in sensory neurons at various levels, including the axon initial segment (174) and the dorsal root ganglion (175), is a driver of neuropathic pain in humans. This could involve both small- and large-diameter nerve fibers. Large-diameter nerve fibers may exhibit spontaneous hyperexcitability, particularly when demyelinated in an inflammatory environment (176). In the peripheral nervous system, this may correspond to conditions of nerve compression, where axons with larger diameter are preferentially affected (177), e.g., in the context of trigeminal neuralgia, carpal tunnel syndrome, or mechanical radiculopathy. Hypersensitivity of large-diameter demyelinated nerve fibers can also occur in the central nervous system, particularly in the context of multiple sclerosis, for example to explain trigeminal neuralgia related to a plaque affecting the trigeminal root entry zone in the pons (178) or Lhermitte's sign triggered by stretching of demyelinated axons of the dorsal columns of the cervical spinal cord (179). Thus, demyelinated Aβ fibers can be the site of ectopic discharges and hyperexcitability causing neuropathic pain presenting as electric shock sensations (180). In these clinical contexts (trigeminal nerve injury, carpal tunnel syndrome, spinal cord injury), abnormalities may be observed in neurophysiological tests, such as delayed blink reflex, slowed sensory conduction of the median nerve, or alterations of somatosensory evoked potentials (181). However, these neurophysiological abnormalities reveal the process of demyelination and not intrinsically the presence of associated pain, which is related to a secondary process of axonal hyperactivity or hyperexcitability. Moreover, even in cases of “nerve compression”, functional axonal hyperexcitability may occur before any structural lesion (such as segmental demyelination) of the nerve fibers, as in classic trigeminal neuralgia related to compression of the trigeminal root by a vessel (182).

In contrast, in nerve compression injuries, small-diameter myelinated (Aδ) and unmyelinated (C) fibers are known to be relatively spared (183, 184) and the PREPs and other small-fiber tests may therefore remain unchanged. Preservation of small-diameter nerve fibers (81) or spinothalamic tract fibers (185) may be associated with some features of neuropathic pain, such as burning sensations. In peripheral nervous system disorder, pure hyperexcitability of Aδ-C fibers may result in neuropathic pain without fiber loss (186), as in primary erythermalgia (187, 188). In the context of spinal cord injury, degeneration of axons within the spinothalamic tract is not at the origin of pain, but may trigger, via inflammation, spontaneous activity in residual neighboring intact axons, which act as “pain generators” and play a crucial role in maintaining central pain (185). Therefore, as in the context of large-diameter fiber system involvement, the detection of structural lesions in the small-diameter fiber system does not establish a direct cause of pain.

Since both large- and small-diameter nerve fibers can be involved in neuropathic pain, whether injured or not, it is not surprising that in patients with diabetic polyneuropathy, the frequency of neuropathic pain does not differ across pure large-, pure small-, and mixed-fiber polyneuropathy (189). This finding was explained by the fact that “in patients with pure large-fiber polyneuropathy, nociceptive nerve terminal involvement might be undetected by standard diagnostic techniques”. This conclusion is incorrect and based solely on the concept that persistent neuropathic pain results from a marked loss of nociceptive afferents, a concept derived from correlational observations that do not prove a causal link (190, 191). This relationship between small fiber loss and neuropathic pain is even counterintuitive, as shown by the fact that neuropathic pain can be treated by local application of capsaicin, which causes intraepidermal degeneration of nociceptive fiber endings (192). Thus, the origin of neuropathic pain is related to excitability disorders that cannot be directly predicted by the type of nerve fiber lesion.

Techniques assessing axonal gains of function, such as genetic testing or microneurography, or peripheral or spinal sensitization, such as RIII reflex recording or secondary hyperalgesia protocols, might be of greater interest for the diagnosis of neuropathic pain. However, a gain of function is not sufficient for the development of spontaneous pain, as discussed in a previous chapter. On the other hand, even though excessive primary afferent input in somatosensory pathways plays a key role in the maintenance of neuropathic pain, central sensitization processes occurring in brain networks, may be responsible for many of the clinical features of chronic neuropathic pain (193, 194). The involvement of such a sensitization of brain networks in neuropathic pain cannot be proven by complementary tests. For example, an increase in the amplitude of PREPs is not specific, and similar brain response enhancement can be elicited by salient but non-painful auditory, tactile and visual stimuli (195). Furthermore, various psychosocial factors may contribute to certain clinical sensory profiles, beyond what can be concluded from the usual interpretation of QST results, for example (196). According to the biopsychosocial model of pain, a large number of complex interactions of psychological and social factors should certainly not be underestimated in patients with chronic neuropathic pain (197). However, these factors escape an approach based only on complementary tests of clinical neurophysiology, skin biopsy or neuroimaging to identify the individuality of pain mechanisms.

## Conclusion

**Figure 2:**
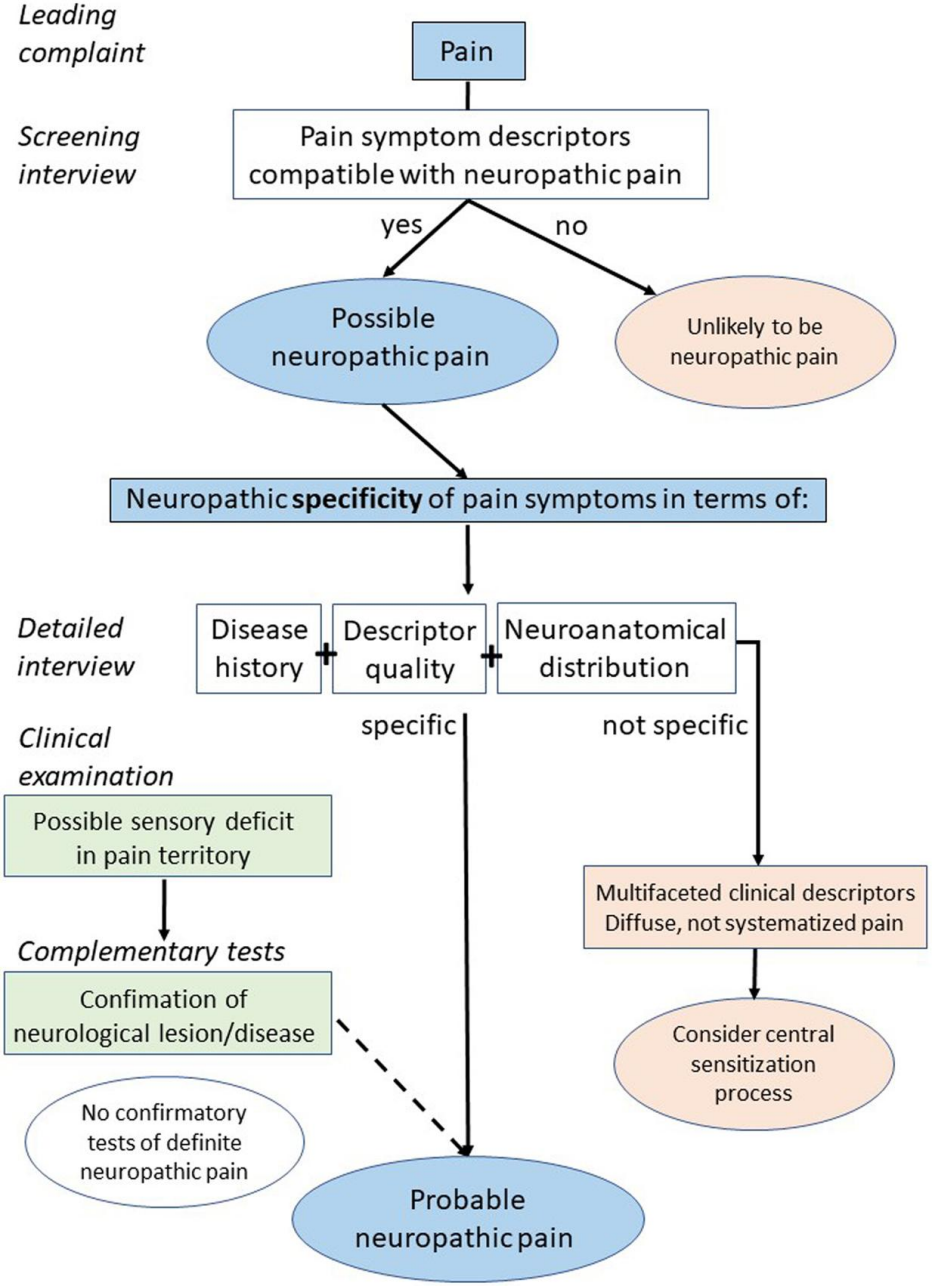
Proposal of a new flow chart for the diagnosis of neuropathic pain.

Sensory deafferentation is a direct cause of sensory loss, not the primary mechanism of neuropathic pain. It is only one of the mechanisms that can lead to secondary phenomena of hyperexcitability/hyperactivity of the somatosensory nervous system, which is the main cause of neuropathic pain. Although complementary tests can identify a structural lesion of the somatosensory nervous system, they are not capable to determine whether or not this lesion is associated with a phenomenon of hyperexcitability/hyperactivity. Indeed, structural lesions of the somatosensory nervous system are not systematically accompanied by neuropathic pain, and neuropathic pain can occur without lesions, in a context of gain of function of sensory nerve fibers. This is the intrinsic reason why complementary tests (clinical neurophysiology, neuroimaging, skin biopsy) aimed at demonstrating the existence of a structural lesion of the somatosensory system, but not a gain of function or changes in neural activities, cannot establish the diagnosis of neuropathic pain. Understanding the pathophysiological mechanisms underlying a pain syndrome relies primarily on the interpretation of the disease history and all clinical signs and symptoms, especially their neuroanatomical distribution and the type of sensory descriptors, but it is not essentially based on or confirmed by complementary tests, as proposed in the algorithm illustrated in Figure 2. In conclusion, complementary tests should be interpreted with caution, because they can formally diagnose a neurological lesion, but not neuropathic pain.

## Data Availability

The original contributions presented in the study are included in the article/Supplementary Material, further inquiries can be directed to the corresponding author.
